# HERV-K Envelope Protein Induces Long-Lasting Production of Autoantibodies in T1DM Patients at Onset in Comparison to ZNT8 Autoantibodies

**DOI:** 10.3390/pathogens11101188

**Published:** 2022-10-15

**Authors:** Marta Noli, Gianfranco Meloni, Stefano Ruberto, Seyedesomaye Jasemi, Elena Rita Simula, Davide Cossu, Marco Bo, Mario Palermo, Leonardo A. Sechi

**Affiliations:** 1 Dipartimento di Scienze Biomediche, Università di Sassari, 07100 Sassari, Italy; 2 Dipartimento di Medicina, Chirurgia e Farmacia, Università di Sassari, 07100 Sassari, Italy; 3 Servizio di Endocrinologia, Azienda Ospedaliera Universitaria, 07100 Sassari, Italy; 4 Struttura Complessa di Microbiologia e Virologia, Azienda Ospedaliera Universitaria, 07100 Sassari, Italy

**Keywords:** HERV-K, *Mycobacterium paratuberculosis*, ZNT8, antibodies, T1DM onset, follow-up, peptides

## Abstract

Human endogenous retroviruses (HERVs) have been thought of as silent passengers within our genomes, but their reactivation has been linked with several autoimmune diseases, including type 1 diabetes (T1DM). In order to evaluate the potential role of HERVs, in addition to the recognized role of HERV-W, we focused on the debated role of the HERV-K family in T1DM. Therefore, we performed a serological evaluation of IgG antibodies against HERV-K Env epitope (HERV-K Env_19–37_) in comparison to an important β-cellular autoimmunity biomarker, ZnT8, from plasma samples of Sardinian children at the onset of T1DM, different T1DM groups (1–5 and 6–12 years since diagnosis), and healthy controls (HCs), by an indirect enzyme-linked immunosorbent assay (ELISA). A significant antibody response was observed against HERV-K Env_19–37_ (*p* < 0.0001) in T1DM patients compared to HCs, and significantly higher IgG responses were detected in the group at the onset compared to the other T1DM groups and HCs. Unlike the trend of the β-cellular autoimmunity autoantibodies, for HERV-K Env antibodies we observed positive values that persist over time up to 5 years since the onset of T1DM. Our results add new evidence about the presence of antibodies against HERV-K in T1DM, but further investigations are necessary to relate these results with the established role of HERVs, considering the contrasting results for HERV-K.

## 1. Introduction

Human endogenous retroviruses (HERVs) constitute approximately about 8% of the human genome. The integrations of the provirus genome into the DNA of germinal cells are the remnants of infections that occurred over several million years, which guaranteed their vertical transmission in a Mendelian fashion [1].

The role of retrovirus in different diseases has remained unknown, particularly for autoimmune diseases that involve a combination of genetic and several environmental factors; HERVs are not infectious but can contribute to the development of inflammatory conditions. A number of factors, such as deletions, mutagenesis, and DNA methylation, can modulate their transcription levels [2]. 

Some diseases are linked to the transcription of certain HERV genes, such as cancer and multiple sclerosis; in addition, the HERV proteins may act as superantigens, which in turn may lead to the development of antibodies against them.

Given their potential pathological effects, HERVs have been proposed as possible cofactors in the etiopathogenesis of various diseases, recently including type 1 diabetes (T1DM) [3,4].

T1DM is one of the most common autoimmune diseases in children and adolescents, caused by the autoimmune response against pancreatic β-cells that results in the local inflammation of pancreatic islets (insulitis) and the progressive loss of pancreatic β-cells, leading to insulin deficiency [5,6]. Both adaptive and innate immunity are involved in the disease process.

Approximately five hundred million adults (20–79 years) are living with T1DM. This number is predicted to rise to 643 million by 2030 and more than 700 million by 2045 [7]. There are several circulating autoantibodies that recognize pancreatic islet antigens: glutamic acid decarboxylase (GAD), islet antigen-2 (IA2), islet cell antibodies (ICAs), zinc transporter 8 (ZnT8), and insulin (IAA) [8]. 

These represent the best biomarkers currently available to identify early immunity and to predict the clinical onset of the disease [9]. ZnT8 is the latest to be identified as a novel autoantigen in T1DM; it may precede the clinical symptoms of the disease and increases sensitivity when analyzed in relation to the other T1DM Abs [10]. Autoantibodies detection before the manifestation of clinical symptoms is a primary diagnostic target for defining the onset of T1DM and may also be useful for the evaluation of immunological therapies, with interventions targeting the autoreactivity of T and B lymphocytes. Different families of HERVs have been associated with T1DM onset as HERV-W, HERV-K. In particular, the most plausible evidence for a functional association with T1DM pathogenesis was found with the HERV-W family [4,11,12]. 

The HERV-W envelope protein (HERV-W Env) has been detected in patients with T1DM and, in particular, in pancreatic acinar cells near the pancreatic lesions of patients with T1DM. HERV-W Env was observed to promote macrophage recruitment within the pancreas and beta-cell dysfunction, as demonstrated by the inhibition of insulin secretion by HERV-W Env in primary cultures of human islets of Langerhans [12]. It was the first study highlighting the detection and the pathogenic properties of HERV-W Env in T1DM.

In previous studies, we portrayed the association of *Mycobacterium avium* subspecies *paratuberculosis* (MAP) and HERV-W in T1DM etiopathogenesis in children from Sardinia, supporting for the first time the hypothesis that it may be possible that both pathogens contribute to the onset of T1DM [13]. Moreover, we highlighted a correlation between anti-HERV-W and proinsulin (PI) autoantibodies in the sera of children with T1DM collected at different times after onset [14].

A different story is associated with HERV-K, where basic questions have been raised regarding the association of T1DM with HERV-K given the contradictory results reported [15,16,17].

In this study, we aimed to investigate the humoral response against HERV-K in association with the detection of classical autoantibodies such as those against ZnT8 in sera from children collected at T1DM onset and different times after diagnosis. 

## 2. Materials and Methods

### 2.1. Sample Collection and Processing

In this retrospective study, samples were obtained from T1DM and healthy controls (HCs) participating in studies. Informed consent was obtained from subjects according to the Institutional Ethical Committee of Sassari University. A total of 70 Sardinian pediatric patients with T1DM were included, including twenty-six patients recruited at the time of the onset. Fifty-seven age-matched controls were recruited for the HCs group during routine check-up visits from the Department of Endocrinology of the University Hospital (AOU) of Sassari. 

Clinical data about the patients and HCs are shown in Table 1.

Blood was taken from each individual using ethylenediaminetetraacetic acid (EDTA) tubes; the blood was processed with Ficoll-Paque^®^ (Sigma-Aldrich, St. Louis, MO, USA) to collect the plasma. Following centrifugation, the resulting supernatant is designated plasma. According to the protocol, the plasma was immediately transferred into a polypropylene tube, slipped into aliquots, stored, and transported at –20 °C. 

### 2.2. Serological Assays 

Serum samples were tested for the presence of Abs against HERV-K Env 19–37 (VWVPGPTDDRCPAKPEEEG) and Znt8 178–186 (MIIVSSCAV) epitopes using indirect enzyme-linked immunosorbent assay (ELISA). All peptides were resuspended in dimethyl sulfoxide (DMSO) and stored in single-use aliquots at −80 °C.

Ninety-six-well Nunc immune-plates were incubated overnight at 4 °C with 0.05 M of carbonate–bicarbonate (pH 9.4, Sigma-Aldrich, St. Louis, MO, USA) and the respective peptides at 10 µg/mL. The plates were incubated for 1 h in a blocking solution with 5% non-fat dried milk (Sigma-Aldrich, St. Louis, MO, USA) and phosphate-buffered saline (PBS), then washed twice in a solution with PBS and 0.05% Tween-20 (PBS-T). Plasma samples were added at a 1:100 concentration and incubated for 2 h at room temperature. After a washing step, each plate was incubated for 1 h with 100 µL of PBS and an alkaline phosphate-conjugated goat anti-human IgG polyclonal antibody (1:1000, Sigma-Aldrich, St. Louis, MO, USA). After washing, each plate was washed in PBS-T and then incubated in Milli-Q water and p-nitrophenyl phosphate (Sigma-Aldrich, St. Louis, MO, USA) for 8–10 min in a dark environment. The optical density was read at 405 nm using a SpectraMax Plus reader (Molecular Devices, Sunnyvale, CA, USA).

Each sample was run in triplicate, and normalization was performed with a positive control setting with the absorbance reactivity at 1.0 arbitrary units (AU)/mL. Negative control sera were also included in all experiments. ELISA precision was determined by calculating both the inter- and intra-assay coefficients of variation. 

### 2.3. Statistical Analysis

Statistical analysis was performed with commercial software GraphPad Prism 9.1 (GraphPad Software, San Diego, CA, USA). Statistical significance was defined as a *p* value < 0.05.

Data distribution was analyzed using the D’Agostino–Pearson omnibus normality test and the Shapiro–Wilk test. Non-parametric data were analyzed using the Mann–Whitney U test and the Kruskal–Wallis test with Dunn’s multiple comparisons test to compare the antibody levels against Znt8 _178–186_ and HERV-K Env_19–37_-derived peptides between the different groups. Receiver-operating characteristic (ROC) was used to choose the cut-off value to assess the sample positivity, which was consequently tested through Fisher’s exact test. A Spearman correlation test was performed among levels of antibodies on HERV-K Env and ZnT8-derived peptides.

## 3. Results

### Prevalence and Titer of ZnT8 and HERV-K Antigens

The humoral responses against ZnT8_178–186_ and the selected highly immunogenic peptide from the envelope of HERV-K Env_19–37_ was carried out by an indirect ELISA assay on the plasma samples of the T1DM and HCs groups. Significant differences were detected between the patient groups and the control group in terms of positivity and mean levels for ZnT8_178–186_ and for HERV-K Env_19–37_.

**Figure 1:**
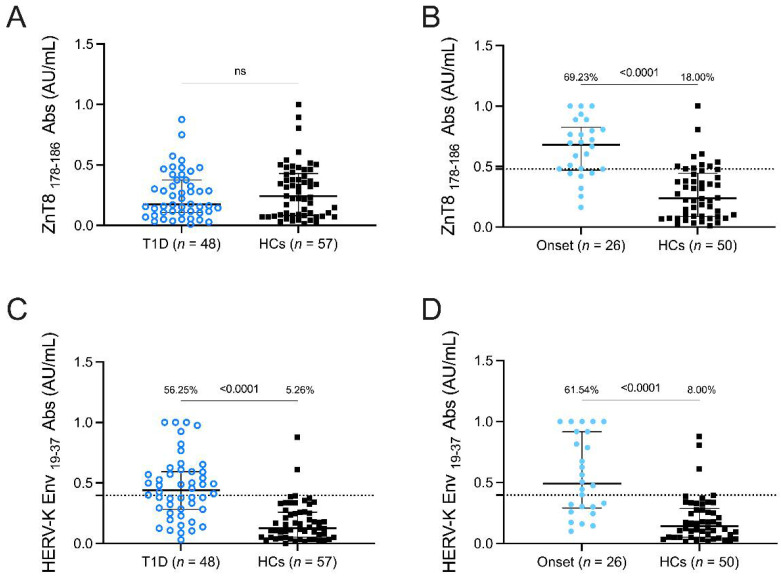
Prevalence of Abs against ZnT8 _178–186_ and HERV-K Env_19–37_ antigens in T1DM children. Plasma samples from T1DM patients, HCs, and patients at onset were tested with indirect ELISA assay. Abs against ZnT8 _178–186_ (**A**,**B**) and HERV-K Env_19–37_ (**C**,**D**) peptides. The dotted lines represent the cut-off values calculated by ROC analysis. The black bars represent median plus interquartile range. The Mann-Whitney *p*-value and the percentage of positive patients’ values calculated by the Fisher’s exact test are indicated in the upper section of the graph.

The positivity and mean levels of anti- ZnT8_178–186_ Abs showed no significant differences between T1DM and HCs patients (Figure 1A) but were significantly higher in patients at T1DM onset (69.23%, 18 out of 26) than in HCs (18%, 9 out of 50) (cut-off value 0.48; AUC = 0.86; *p* < 0.0001 Figure 1B).

Concerning HERV-K peptide, we found for the first time a significant difference in the antibody response between T1DM and HCs and at T1DM onset (Figure 1C,D). In T1DM patients, 27 out of 48 were positive (56.25%) in comparison to HCs (5.26%), where 3 out of 57 were positive (cut-off value 0.40; AUC = 0.84; *p* < 0.0001 Figure 1C). In the onset group, 16 out of 26 were found to be seropositive to HERV-K Env_19–37_ (61.54%), whereas only 4 out of 50 were positive in HCs (8%,) (cut-off value 0.39; AUC = 0.84; *p* < 0.0001 Figure 1D). 

To deepen the significance of the results regarding of the HERV-K Env_19–37_ humoral response, we decided to stratify the population to investigate a potential change in humoral response related to disease duration and in relation to the trend of the autoantibody against ZnT8_178–186_. The results obtained from testing the four groups investigated (onset, 1–5 yr, 6–12 yr, HCs) were analyzed by Kruskal–Wallis test and using Dunn’s post hoc analysis.

**Figure 2:**
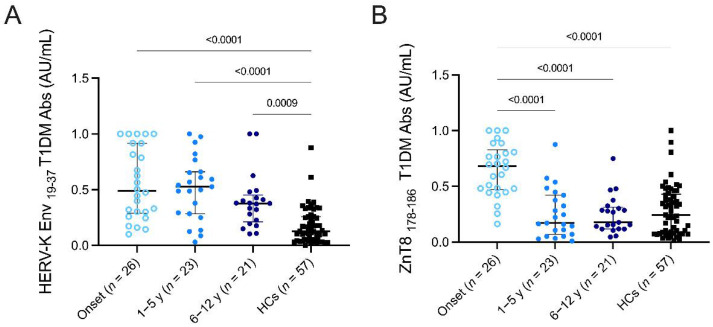
Prevalence of Abs against HERV-K Env_19–37_ and ZnT8 _178–186_ antigens in T1DM children. Stratification of T1DM patients (onset, 1–5 years, 6–12 years) and HCs were tested against HERV-K Env_19–37_ (**A**) and ZnT8_178–186_ (**B**) peptides. Kruskal–Wallis *p*-values are indicated in the upper part of each graph. The median value plus the interquartile range is represented by the black bars.

The results exhibited statistically significant differences in the antibody responses against HERV-K Env_19–37_ in the different groups (Figure 2A) between patients at onset and HCs (*p* = < 0.0001), 1–5 yr and HCs (*p* = < 0.0001), and between the group 6–12 and HCs (*p* = 0.0009). Significant differences were also found in the antibody responses directed against ZnT8 _178–186_ (Figure 2B) between patients at onset, the 1–5 yr group (*p* = < 0.0001), 6–12 yr (*p* = < 0.0001), and HCs (*p* = < 0.0001), confirming its specificity as a biomarker for the disease.

**Figure 3:**
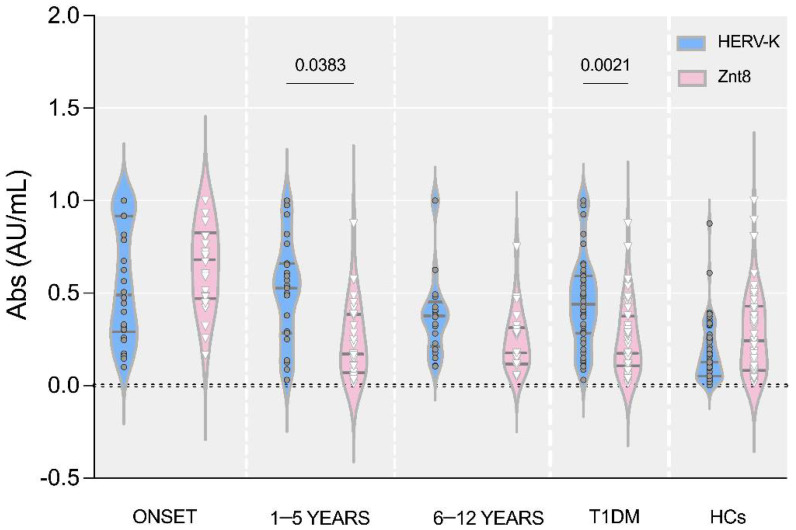
Violin plot representations of the antibody response against HERV-K Env_19–37_ compared to ZnT8 _178–186_ in the stratification of T1DM patients (onset, 1–5 years, 6–12 years) and HCs.

The analysis of the antibody response directed against HERV-K Env_19–37_ in comparison with the trend of self-antibody ZnT8_178–186_ showed statistically significant differences for the groups 1–5 yr (*p* = 0.038) and for the group with the total diabetic population (Figure 3).

**Figure 4:**
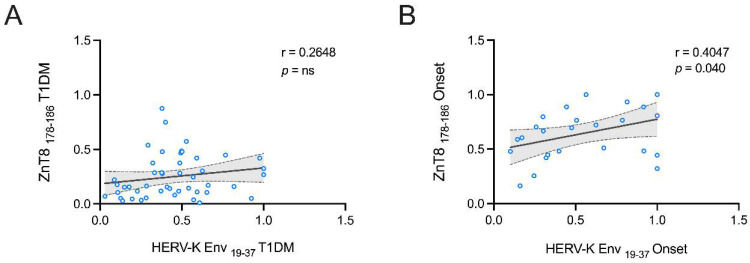
Scatter plot expressing the correlation between ZnT8 _178–186_ and HERV-K Env _19–37_-derived peptides in a pediatric population with T1DM (**A**) and at onset (**B**).

An additional analysis was carried out to evaluate possible correlations between anti-ZnT8 _178–186_ and HERV-K Env_19–37_ humoral responses. Spearman’s correlation shows that in T1DM patients, ZnT8_178–186_ plasmatic levels are not correlated to the humoral response against HERV-K Env-derived peptides (Figure 4A), but for the group at the onset, we found a positive correlation between ZnT8 _178–186_ and HERV-K Env_19–37_ (r = 0.40 *p* < 0.0001, Figure 4B).

## 4. Discussion

T1DM is considered an autoimmune disease, caused by immune responses in genetically susceptible individuals, characterized by T cell infiltration against pancreatic b-cells, resulting in their destruction and marked by the production of Abs. The first objective of this study was to determine the humoral response against HERV-K Env_19–37_. A further aspect investigated was the change in the prevalence of these Abs at the diagnosis of the disease and at different years since diagnosis, and whether these changes follow a trend referable to an important β-cellular autoimmunity marker, such as ZnT8, which plays an important role in the progression of the disease and which has been identified as one of the main biomarkers for type 1 diabetes. These Abs are present months or years before the clinical manifestations [18,19]. ZnT8 is a protein encoded by the SLC30A8 gene on chromosome 8 that is expressed selectively on the insulin granular membrane of pancreatic b-cells; its function is to maintain the zinc balance in beta cells by importing and exporting zinc ions [20,21]. It plays a key role in the regulation of insulin signaling and glucose homeostasis, and it is also involved in insulin synthesis, storage, and secretion in humans [22].

In addition, ZnT8-derived peptides have been identified as important biomarkers of diabetes autoantigens that may trigger a self-reactive CD8+ T lymphocytes response, suggesting that this antigen plays an important role in the progression of the disease [23]. 

The secretion of insulin granules increases the exposure of the autoantigen ZnT8 to the cell surface, which can initiate ZnT8 epitope-specific T cell-mediated beta cell destruction, resulting in T1DM.

This mechanism is amplified when beta cells are destroyed, as this increases the release of autoantigens, which thus intensifies the T cell-mediated immune response. 

Several peptides of Znt8 were previously identified by our group, which share more than 50% of their identity with *Mycobacterium avium* subspecies *paratuberculosis* (MAP). The potential involvement of MAP in diabetic pathology may result from molecular mimicry with the ZnT8 _178–186_ epitope, which leads to autoimmune responses. The cross-reactivity to common target sequences and the specificity of anti-MAP-ZnT8 Abs has been verified by competition assays in previous studies [24]. 

It is known that the innate immune cells can be specifically activated by the envelope surface proteins of HERVs through the pattern recognition receptors CD14 and TLR4 [25].

Among the different HERV families, the largest evidence of a functional association in the pathogenesis of T1DM has been presented for HERV-W Env [12].

This association was also confirmed by our group. HERVs-encoded proteins may be the principal trigger of the disease when they are expressed or overexpressed. It is possible that these proteins could induce the overproduction of anti-HERVs antibodies by B lymphocytes with a protective role, as observed in ALS recently [26]. Several studies observed the detection of HERV-K Env in the cerebrospinal fluid (CSF) and brain in amyotrophic lateral sclerosis (ALS) patients, noting its specific neurotoxic effect in vitro and in vivo [27,28,29]. Significantly increased levels of Abs targeting HERV-K Env in ALS patients were initially reported by our group [26,30].

Conflicting opinions regarding the role of HERV-K in T1DM have been published [15,16]. Past studies based on viral sequence expression IDDMK1 [31,32] initially in favor of such a link have subsequently excluded the association between HERV-K and T1DM [15,16].

Although HERV-K association has not been validated for T1DM, we wanted to investigate the presence of IgG antibodies against HERV-K _19–37_ and ZnT8 _178–186_ in order to better understand the role of antibodies against different HERVs at onset and how these evolve during disease progression. This study showed a high seroprevalence of anti- HERV-K Env_19–37_ Abs in pediatric patients at T1DM onset. Of note, in contrast to antibodies against HERV-W, we observed a constant Abs response against HERV-K Env_19–37_ that remains over time, up to 5 years of disease, and then decreases. HERV-K is probably not involved in the pathogenesis of T1DM but is related to a general HERV-K expression in other inflammatory conditions; however, this has to be confirmed [33,34,35].

The marked decline of ZnT8 already 1 year after onset probably reflects a reduction in these autoimmune processes over time due also to the lack of the antigen. 

In conclusion, our results provide further evidence of the association of antibodies against HERVs and autoantibodies and lead us to question if these autoantibodies against HERV-W and HERV-K may be useful biomarkers in T1DM progression. 

Further studies are needed to clarify the role of HERV-K, as well as the diagnostic and prognostic value of serum Abs, in order to have a more accurate picture of disease. Additional investigations on the expression levels of the HERV-K envelope protein in PBMCs might be useful.

## Figures and Tables

**Table 1:** Demographic and clinical characteristics of patients and healthy controls.

Study Population		Female	Male	Mean Age ± SD
T1DM (n = 70)	Onset	9	17	7.66 ± 4.70
	1–5 years	10	13	11.48 ± 3.52
	6–12 years	6	15	15.03 ± 3.90
Healthy controls (n = 57)		33	24	10.5 ± 4.21

## Data Availability

Not applicable.
